# The Relationship between Social Norms, Avoidance, Future Orientation, and Willingness to Engage in Climate Change Advocacy Communications

**DOI:** 10.3390/ijerph182413037

**Published:** 2021-12-10

**Authors:** Carl Latkin, Lauren Dayton, Catelyn Coyle, Grace Yi, Da-In Lee, Abigail Winiker

**Affiliations:** 1 Department of Health, Behavior and Society, Bloomberg School of Public Health, Johns Hopkins University, Baltimore, MD 21205, USA; ldayton2@jhu.edu (L.D.); ccoyle5@jhu.edu (C.C.); gracetyi95@gmail.com (G.Y.); awinike1@jhmi.edu (A.W.); 2 Division of Infectious Diseases, Johns Hopkins University School of Medicine, Baltimore, MD 21205, USA; 3 Krieger School of Arts & Sciences, Johns Hopkins University, Baltimore, MD 21218, USA; dlee227@jhu.edu

**Keywords:** climate change action, social norms, online, social media, advocacy, avoidance, health communications

## Abstract

This study examined factors associated with willingness to engage in communication behaviors related to climate change advocacy. Data were collected as part of an online, longitudinal US study beginning in March 2020. Outcomes included willingness to post materials online, contact state legislators, and talk with peers about climate change. Covariates included climate change-related social norms, avoidance of climate change information, and perceptions of the future impact of climate change. A minority of the 586 respondents (23%) reported regular conversations about climate change, while approximately half of the respondents reported willingness to discuss climate change with peers (58%), post materials online (47%), and contact state legislators (46%). Strong predictors of willingness to engage in each climate change communications behaviors included climate change social norms, not avoiding climate change information, and believing that climate change will have a negative impact on the future. Findings indicate the importance of designing programs to foster increased climate change communications in order to promote community-level climate change advocacy norms.

## 1. Introduction

The current and future impacts of climate change on public health and well-being are profound, and a large proportion of the global population perceives climate change to be an important issue [1,2,3]. Understanding effective communication strategies about climate change is critical [4]. For example, research suggests that simple and brief educational videos can enhance understanding of climate change [5,6]. Several studies have also documented that varying the ways in which climate change is framed and presented can change attitudes, though partisan predispositions may influence the impact of this framing [7,8,9,10,11,12]. Changing attitudes through effective communications about climate change may not lead to behavior change, as the link between attitudes and behaviors is often tenuous [13,14]. Indeed, attitudes and risk appraisals tend to only account for a small amount of the variance in most analytic models predicting behavior change [15,16]. However, attitudes about a specific behavior are much more predictive than more general attitudes [17,18]. Thus, in the current study, we assess attitudes about specific climate change communications and actions, including posting climate advocacy materials on social media, lobbying policymakers, and talking to important others about climate change.

The question of which individual-level behavior changes can lead to the measurable mitigation of climate change is of critical importance. Voluntary, individual-level behaviors such as recycling, reduced energy usage, and switching energy sources to electric from gas are likely to have only a modest impact on the climate change trajectory, if one at all [19,20]. Such behaviors can also be problematic if people believe that these actions alone will be effective in addressing climate change. On the other hand, an individual’s adoption of these voluntary behaviors to address climate change may enhance their social identity as someone concerned about climate change, consequently leading to increased interest and engagement in political and policy actions that may have a more sustained impact [21]. Research also suggests that involvement in one type of political action can lead to involvement in other types [22,23]. Consequently, facilitating engagement in modest climate change action may lead to greater general political involvement or climate-related action. Some theoretical approaches have integrated individual-level behaviors with political and cultural factors that serve as barriers to pro-environmental behaviors [24]. The key to addressing climate change is advancing major structural changes in the amount and types of energy use [25,26,27]. However, what are the most effective methods to foster governmental policies that might mitigate climate change? One approach is the direct lobbying of policymakers and political leaders to prioritize meaningful climate change legislation and policies. Political action can also be used to elect individuals who are committed to addressing climate change. To garner sufficient political pressure, it is also imperative to develop effective communication strategies to focus public opinion in order to demand that policymakers develop and implement meaningful climate change legislation and policies.

Communications processes are integral to changing public opinion. Research indicates that family and friends are some of the most important sources of climate change information [28]. Although one study found climate change to be an infrequent topic of conversation [29], there are mixed findings on a “spiral of silence” around climate change. The “spiral of silence” refers to the tendency for individuals to withhold minority opinions for fear of social isolation [29,30]. Furthermore, perceptions of support for environmental issues in one’s social group, as well as social identity, influence pro-environmental engagement [31]. Peer communication about climate change is therefore an important area of study, as talking about global warming can lead to greater acceptance of climate science [32]. However, little is known about individuals’ interests in communicating to family or peer group members about climate change or lobbying legislators, or factors that contribute to willingness to engage in climate change advocacy communication.

Social norms may contribute to a lack of discussion of climate change and the “spiral of silence”, as individuals may perceive there to be a proportion of their peer group that objects to their behavior. There is a wealth of evidence that social norms can influence environmental behaviors such as electrical consumption and recycling [33,34,35,36,37]. However, there is little information about how norms may impact climate change conversations or advocacy. If there are normative expectations that climate change is not an appropriate subject of conversation, people may therefore assume that it is also an inappropriate topic for advocacy. Further, even if most peers are seen to support an individual’s views, they may nonetheless be influenced by norms around avoiding discussion of controversial topics such as climate change. A recent systematic review of research on climate change in social factors and psychology publications highlights the need for a greater understanding of the influences of social network members on climate change behaviors [38]. In the current study, we examined factors associated with the willingness to talk to friends about climate change and post materials on social media, which is often viewed by network members.

An individual’s motivated reasoning and self-efficacy may also contribute to their willingness to engage in climate change advocacy communication. There is substantial literature on motivated reasoning or motivated cognitions and climate change behaviors [39,40,41]. This literature is based on the notion that those who are climate change skeptics or who downplay the severity of climate change are motivated to spend less time reading or viewing materials on climate change because that information may lead to them questioning their beliefs. Another way of understanding these cognitions is that the issue of climate change is seen as less of a priority, which may be due to low media coverage or the perception that it is not an important issue in one’s peer group. Consequently, less time may be spent on obtaining and processing information about causes and consequences of climate change as well as how to address it. Another dilemma with trying to address climate change is the magnitude of the issue. People may not believe that they have the ability to help mitigate climate change. This lack of personal self-efficacy could lead to inaction [42].

The limited body of research on climate change advocacy communication has focused on how to convince people that climate change is caused by humans and that mitigation efforts can successfully reduce its deleterious impact. There is less literature on fostering and facilitating climate change collective actions that could lead to meaningful policy changes. In recent research, a study by Bury et al. found that hope was associated with support for climate change actions, and in Canada, Smith et al. documented that social norms and perceived risk were linked to willingness to vote against politicians who do not support policies that reduce climate change and support of a $20 monthly tax to aid efforts to adapt to climate change in their community [43,44].

In the current online longitudinal study, we examined the frequency of individuals’ current climate change discussions and modeled willingness to engage in climate change communications and behaviors. We assessed willingness to (1) talk to family and friends, (2) contact legislators, and (3) post materials online about climate change. We then modeled these three outcomes and examined potential barriers, which included avoidance of climate change information, perceptions of futility in effecting change, self-efficacy to tackle climate change, and social norms, including perceived negative reactions from friends. We hypothesized that social norms would be a strong driver of willingness to post materials, talk to family and friends, and contact legislators about climate change. We also anticipated that the individual-level factor of avoidance of climate change news would be negatively associated with willingness to engage in climate change actions. We additionally conducted a sub-analysis to examine perceptions of whether peer support or discouragement might have a stronger association with willingness to engage in climate change activities.

## 2. Methods

Study respondents, who were adults in the US, participated in an online longitudinal study that began in March 2020. This study aimed to examine individual, social, and societal-level fluctuations amidst the COVID-19 pandemic’s rapidly changing landscape. Study periods occurred every few months and sought to capture changes in scientific knowledge of infection, the extent of infectious spread, and progress in vaccine development. All data for the analyses presented in this paper were from the fourth wave except for the demographic and political affiliation data, which were collected at baseline. All respondents who successfully completed the first survey were invited to participate in the subsequent rounds of data collection. Study respondents completed the first survey between 24–27 March 2020, and the fourth wave of the study was administered from 18–28 November 2020.

Study participants were recruited through Amazon’s Mechanical Turk (MTurk) service. This approach is regularly used by health researchers, as it allows for a diverse sample to be collected in a rapid and timely fashion [45]. Prior research has indicated that MTurk provides better quality data in less time than other methods for recruiting convenience samples [46]. Study populations recruited through MTurk are not nationally representative but have been documented to outperform other opinion samples on several dimensions [47]. Studies using MTurk have also demonstrated good reliability [48]. Study protocols followed MTurk’s best practices for research, which included ensuring participant confidentiality, protecting study integrity, generating unique completion codes, integrating validity-checks throughout the survey (which included low probability events such as frequency of deep sea fishing in Alaska and number of appendages that had been removed) and repeating study-specific qualification questions [46,49,50].

The demographic characteristics of MTurk appear to be stable [51], and although MTurk respondents are more liberal than the general public, those who are conservative do not significantly differ in their attitudes from conservatives recruited by other sources [52]. Eligibility for the study included being age 18 or older, living in the United States, being able to speak and read English, having heard of the coronavirus or COVID-19, and providing written informed consent. To enhance study validity, eligible participants had to pass attention and validity checks embedded in the survey. Participants were paid $4.00 for the fourth survey, which was equivalent to approximately $12 per hour. The study protocols were approved by the Johns Hopkins Bloomberg School of Public Health Institutional Review Board.

## 3. Measures

The key outcome measures of climate change conversations and advocacy included the following three survey items: (1) “I would be willing to post materials online about climate change”, (2) “If trained, I would be willing to talk to my friends about climate change”, and (3) “If trained, I would be willing to contact my state legislators about climate change”. The response categories were: “Strongly agree”, “Agree”, “Neither agree nor disagree”, “Disagree”, and “Strongly disagree”. These questions were dichotomized such that “Strongly agree” and “Agree” responses were compared to those who responded, “Neither agree nor disagree”, “Disagree”, or “Strongly disagree”. We assessed “state legislators” rather than national legislators because some national environmental organizations send emails and social media posts that only require inserting your name and clicking an icon to send an email to national legislators. We did not include rallies or protests due to the timing of the survey, which was at the height of the COVID-19 pandemic.

Prior research suggests that political ideology, social norms, and motivated reasoning are linked to environmental and climate change attitudes and behaviors [11,36,37,40]. We wanted to examine if these factors are also linked to climate change actions. Climate change social norms were based on measures developed by Doherty and Webler [53] and assessed with the following questions: (1) “If I were to talk to most of my friends about the impact of climate change, they would be uncomfortable”, (2) “Most of my friends would disapprove if I were to post things on social media on climate change”, and (3) “Most of my friends think that climate change is a major issue”. These three questions had the response categories of “Strongly agree”, “Agree”, “Neither agree nor disagree”, “Disagree”, and “Strongly disagree”. These items were added together to generate a scale of social norms, where each “Strongly agree” response received a value of 1, and each “Strongly disagree” response received a score of 5. The third climate change social norms question was reverse coded so that higher scores on the scale reflected stronger social norms of supporting climate change science (Cronbach’s alpha = 0.70, Range 3–15, Median = 11, Mean 11.28, SD = 2.44).

Two questions assessed avoidance of the topic of climate change: (1) “I tend to avoid news on climate change”, and (2) “With everything else going on in the world, I don’t have much interest in climate change”. The response categories were “Strongly agree”, “Agree”, “Neither agree nor disagree”, “Disagree”, and “Strongly disagree”. These questions were added together for a measure of climate change avoidance. (Cronbach’s alpha = 0.79, Range 2–10, Median = 8, and Mean 7.53, SD = 2.17).

The future impact of climate change was assessed by the item: “The future of many young people will be much worse due to climate change [32]. Perceived self-efficacy to address climate change was assessed with the item: “There is nothing I can do about climate change”. The response categories were “Strongly agree”, “Agree”, “Neither agree nor disagree”, “Disagree”, and “Strongly disagree”. The item, “How concerned are you about climate change”? included the response categories of “Great deal”, “Quite a bit”, “Some”, “Very little or None”. The survey question, “How often do you talk to your friends and family about climate change”? included the response categories of “Every week”, “Several times a month”, “A few times a year”, and “Never”.

Political ideology was assessed with the standard question: “Where would you place yourself on a 1 to 7 scale ranging from “Very liberal” to “Very conservative”? The response categories were “Very Liberal”, Liberal”, Slightly Liberal”, “Moderate”, “Slightly Conservative”, “Conservative”, and “Very Conservative”. There were three individuals who did not provide data for this question and were assigned a median value of 3.

Gender, education, and income were also assessed. The level of education was collapsed to reflect some college or less versus bachelor’s degree or higher. Income was dichotomized at the median of $60,000 or below. The response categories for self-reported race/ethnicity included “White”, “Black”, “Asian”, “Hispanic”, “Mixed”, or “Other”. Due to sample size, the categories of Hispanic, mixed, and other were collapsed. The demographic variables were collected at the first wave.

## 4. Analysis

Descriptive statistics of means, standard deviations, and percentages were first calculated, and then bivariate logistic regression models were used to examine the dichotomous outcomes. Three multivariable logistic models were employed to assess the differences in willingness to (1) post materials, (2) contact state legislators, and (3) talk with peers about climate change. These multivariable models controlled for all the other variables to examine the independent contribution of each independent variable. This approach allows for both the measurement and the control of potential confounds. For the multivariable logistic regression models, all sociodemographic variables (age, sex, income, race/ethnicity, and education) were included as potential confounders, and other variables with a p-value of <0.20 in the bivariate models were also included in the adjusted model [54,55]. In a final analysis, we examined the individual social norm items to assess whether perceived negative peer response to climate change actions would have the same impact as positive attitudes of peers. The sample size was not determined for the specific analyses; rather, the goal was to obtain a sample for the longitudinal study and have sufficient power to detect a 15% difference with 500 respondents.

## 5. Results

### 5.1. Baseline Characteristics

Characteristics of the 586 participants who completed waves 1 and 4 of the MTurk study are presented in Table 1. Overall, the mean age was 39.6 years (SD = 11.7 years), 57.3% of the respondents were female, 55.6% had at least an undergraduate degree, and 45.6% had an annual income >$60,000. Eighty-one percent of the participants were white, followed by 12.6% who self-identified as “other”, and 6.3% were non-Hispanic Black. Regarding climate change, roughly two-thirds of the participants reported that they were a great deal or quite a bit concerned about climate change. The same percentage also believed that the future of young people would be worse due to climate change (67.8%) and that their actions could address climate change (64.3%). However, 77.3% indicated that they only talk about climate change to friends and family a few times a year or never, 19.8% reported talking about climate change several times a month, and 2.9% reported talking about climate change weekly.

### 5.2. Climate Change Behaviors

Less than half of the respondents stated that, if trained, they would post materials about climate change online (47.1%). In bivariate analysis, self-perceived efficacy to address climate change was positively associated with posting climate change materials, and political ideology was negatively associated with this outcome. However, these relationships did not retain significance in multivariate models (Table 2). In both bi- and multivariate analysis, we found that the odds of posting climate change material online increased 25% (aOR: 1.25, 95% CI: 1.14–1.41) for each unit increase on the climate change social norms measurement scale (Table 2). Odds of willingness to post materials online were also higher among respondents who did not avoid climate change material (aOR: 1.27, 95% CI: 1.10–1.46) and agreed the future of many would be worse because of climate change (aOR: 1.54, 95% CI: 1.19–1.99). Reporting an annual income of more than $60,000 (aOR: 0.52, 95% CI: 0.35–0.79) was associated with reduced odds of willingness to post climate change materials (aOR: 0.52, 95% CI: 0.35–0.79). The results presented in Table 2 suggest that in most of the models, the demographic variables were not significantly associated with the three outcomes. However, in the multivariable models, respondents with lower household incomes were significantly more willing to post materials on climate change than those with higher incomes, and those respondents with less education reported a greater willingness to talk to friends about climate change.

As with posting climate change materials, less than half of the respondents said they were willing to contact their state legislature regarding climate change (45.9%). In bivariate analysis, self-perceived efficacy to address climate change was positively associated with contacting state legislators; however, this variable did not retain significance in adjusted models. In both unadjusted and adjusted models, participants’ willingness to contact state legislators about climate change was associated with climate change social norms (aOR: 1.12, 95% CI: 1.01–1.24), not avoiding climate change information (aOR: 1.40, 95% CI: 1.21–1.63), and worry about the future impact due to climate change (aOR: 1.63, 95% CI: 1.26–2.13). The odds decreased among participants with conservative political beliefs (aOR: 0.85, 95% CI: 0.74–0.96).

**Figure 1:**
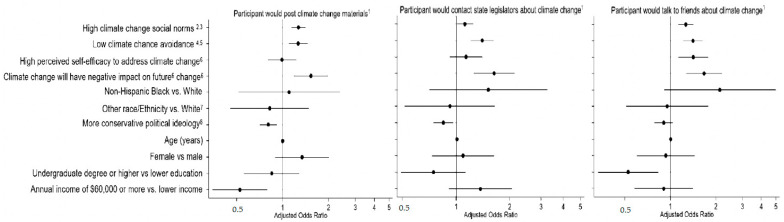
Visual Depiction of Adjusted Odds Ratios of Predictors of 3 Forms of Climate Change Communication Behaviors from 586 Participants who completed the MTurk Surveys at Waves 1, and 4. ^1^ Logistic regression with “no” as reference group; ^2^ Climate change social norms scale: 3 (low)-15 (high); ^3^ Calculated by summing the responses to the following items: (1) If I were to talk to most of my friends about the impact of climate change, they would be uncomfortable, (2) Most of my friends would disapprove if I were to post things on social media on climate change, (3) Most of my friends think that climate change is a major issue. Response options are: 1-strongly agree, 2-agree, 3-neither agree nor disagree, 4-disagree, 5-strongly disagree; ^4^ Climate change avoidance scale: 2 (tends to avoid), 10 (does not avoid); ^5^ Calculated by summing responses to the following items: (1) I tend to avoid news on climate change, and (2) With everything else going on in the world, I don’t have much interest in climate change. Both items have the response options: 1-strongly agree, 2-agree, 3-neither agree nor disagree, 4-disagree, 5-strongly disagree; ^6^ Responses options: 1-strongly agree, 2-agree, 3-neither agree nor disagree, 4-disagree, 5-strongly disagree; ^7^ Includes Hispanic, Asian, Mixed, Other; ^8^ 1-very liberal, 2-liberal to conservative, 3-slightly liberal, 4-moderate, 5-slightly conservative, 6-conservative, 7-very conservative.

Almost 60% of respondents said they would discuss climate change with friends and family. Political ideology was associated with willingness to communicate with peers about climate change in unadjusted but not adjusted models. Like the other two climate change behavioral outcomes, the odds of talking with friends and family were significantly higher with increasing climate change social norms (aOR: 1.27, 95% CI: 1.13–1.42), if a participant did not avoid information about climate change (aOR: 1.41, 95% CI: 1.22–1.64), if a participant believed they could help address climate change (aOR: 1.42, 95% CI: 1.13–1.78), or that the future impact of climate change would be worse (aOR: 1.67, 95% CI: 1.28–2.20). The pseudo R^2^ values were 0.254, 0.335, and 0.242 for our outcomes of willingness to post materials, discuss climate changes with friends, and contact legislature, respectively. Figure 1 presents the visual results of the odds ratios found in Table 2 with the adjusted multivariable items.

Finally, we assessed the individual social norm items and examined whether perceived negative peer response to climate change actions had the same impact as perceived positive attitudes of peers. The results (Table 3) suggest that perceived negative responses did not have a greater association than the positive norms with the outcomes of willingness to engage in climate actions. The largest Chi-square values were consistently found for the associations with the survey item “most of my friends think that climate change is a major issue”.

## 6. Discussion

The current study addresses the question of who is and how people are willing to communicate with their peers about climate change. The study responds to Tam and colleagues’ call for greater attention to studying the influence of peers on climate change behaviors [38]. Study findings identified that few respondents have frequent discussions about climate change with family and friends, and only 23% reported such conversations several times a month or more. This finding coincides with responses reported in the 2020 Yale Climate Change and the American Mind survey, which asked the question, “How often do you discuss global warming with your family and friends”? Only 36% of respondents reported often or occasionally, while the majority (64%) reported rarely or never [56].

However, these data suggest that there is a potentially large group of people who, if trained, are willing to engage in climate change communication actions, especially with their social network members. Over half (58%) of respondents reported that, if trained, they would be willing to talk to friends about climate change. A slightly different question about willingness to talk to family and friends about climate change, which did not include the component about training, indicated that 35% were very willing and 46% were somewhat willing. This level of willingness to initiate conversations was substantially higher than the actual conversation frequency. This gap between willingness to initiate conversations about climate change and the frequency of those conversations suggests an untapped potential for many more individuals to become involved in climate change communication behaviors. Receiving specific training and support may help overcome the identified gap between willingness to communicate about climate change and engaging in the behavior.

The current study also identified that social norms and individual-level factors are associated with individuals’ willingness to engage in climate change communication actions. Unsurprisingly, those who tended to avoid news about climate change and perceived it to be less of a priority were less interested in posting material or talking to friends. If the majority of those concerned about climate change are willing to take action by talking to family and friends, some of whom may avoid climate change news, these conversations may result in greater engagement among those who tend to avoid the topic. Furthermore, our study found that increased social norms surrounding climate change discussions was significantly correlated with willingness to talk to others about climate change. Initiating climate change conversations with friends and family may also work in a reciprocal fashion, whereby friends and family who engage in such discussions begin to perceive greater social norms that support climate change-related conversations and become more likely to initiate conversations of their own. In terms of social norms, there was no evidence that concerns about peers disapproving of climate change actions had a greater impact on willingness to engage in addressing climate change activities than perceptions of peers being concerned about climate change. This finding suggests that encouraging individuals to communicate with their peers about climate change can reach both individuals who believe in climate change as well as those who deny it. By reaching this wider audience, communication between peers may enhance social norms and hence help sustain climate action behaviors as well as potentially change beliefs through conversations with climate change deniers. However, our measures of types of social norms were limited, and future research should examine in greater detail which norms may support or impede climate change action.

Findings from this study also suggest that there were few statistically significant associations with age, sex, educational stage, and ethnicity. However, it is interesting to note that respondents with lower household incomes were significantly more willing to post materials on climate change than those with higher incomes, and those respondents with less education reported a greater willingness to talk to friends about climate change. A more conservative political ideology was associated with a reduced willingness to engage in climate change advocacy actions. These findings suggest that climate change advocacy efforts must frame messages to attract bipartisan supporters.

We also observed that avoidance of the topic of climate change was negatively associated with willingness to engage in climate change action. This association may be due in part to motivated reasoning, yet it is also likely that people avoid the topic because they feel helpless to address it. Our findings did suggest that self-efficacy is linked to willingness to engage in climate change activities, and hence messages seeking to engage the public in climate change actions should consider targeting self-efficacy to address climate change.

One communications approach to increase engagement in climate change action was used by the #MakeItBetter campaign [57]. The #MakeItBetter campaign was developed by the Ontario Public Health Association as a health-focused climate communication strategy with the goal of increasing public support for climate action [57]. The campaign promoted community-building resources, highlighted community assets, and encouraged activism, such as participating in policy discussions at the provincial level. The campaign emphasized the availability of actions to protect families and communities from climate change and encouraged social discourse about climate change. While there is no published outcome evaluation of the campaign to date, this type of approach of encouraging conversations, political action, development of local groups, and enhancement of social identities for climate change action is highly promising.

To ensure that willingness to engage in climate change advocacy turns into action, there is a need to reduce barriers to sharing effective climate change materials and further provide support and positive feedback for those who do engage in climate action. Moreover, individuals need to be exposed to messages that foster self-efficacy to enhance their belief that they can address climate change. Further campaigns may provide individuals with options and communication skills to become change agents.

Many political action organizations focus on encouraging their members to contact legislators about issues of concern and lobby for certain bills or other legislative actions. Although these actions are critical, they may not lead to changes in public support or in perceived social norms, since these lobbying efforts are often not seen by the public. Some online lobbying approaches try to reduce the effort needed to contact legislators by allowing individuals to simply click on a website that is prepopulated with information to forward. However, this approach may not lead to an increase in climate change conversations. Climate change advocacy organizations may thus wish to consider developing methods to encourage and train members to talk to family and friends about climate change. Brief YouTube videos and simple trainings could be used to provide individuals with instructions on how to broach conversations on climate change topics, especially where there may be concerns about respondents’ reactions. These trainings may also include information on how to enhance and maintain discussions on topics or questions infrequently discussed. Such trainings coupled with unobtrusive reminders and positive feedback may also help to facilitate climate change conversations. Future research should examine factors that lead to increased and sustained climate change conversations among social network members.

The current study’s limitations should be noted. As with prior studies with similar sampling strategies, this sample was over-representative of Democrats, as assessed by political party affiliation. However, prior research using MTurk suggests that responses by conservatives are similar to those found in other samples of conservatives [52]. We also did not assess the climate change-related behaviors of climate change political actions, donations to organizations that address climate change, or emailing others about climate change. Prior research indicates that current climate actions among the American population are very low (1%), and hence we would have insufficient statistical power to examine the actual behaviors as outcomes [58]. One domain that we did not examine was the impact of exposure to educational materials, such as lectures, on climate change. These materials are readily available, and the COVID-19 pandemic has increased the availability of video conferences and online lectures. These materials may also provide individuals information and guidance for climate change actions and link them to organizations that are engaged in climate change advocacy. The current findings fit with Reid’s schematic on climate change education, which highlights the importance of developing practical skills and promoting community engagement to address climate change [59].

Although not a random sample, the findings suggest that a large number of adults in the US are willing to talk to family and friends, contact state legislators about climate change, and post materials online about climate change. These findings demonstrate a need to develop effective training and methods to recruit potentially interested individuals to lobby legislators directly and indirectly, through public opinion, to encourage policymakers to pass meaningful climate change legislation and replace those resistant to addressing the climate change emergency. However, individual-level factors may not in themselves lead to greater climate change actions unless there are organizations and social structures that can tap into individuals’ willingness to engage in climate change actions.

These limitations notwithstanding, the high level of willingness to engage in climate action expressed by study respondents is promising and suggests that there is a largely untapped reservoir of potential for climate actions on an individual level. The study findings expand our understanding of the potential influence of social norms on pro-environmental behaviors and the potential role of intrapersonal factors in climate change advocacy. Therefore, in framing efforts to encourage climate change action, attention should be paid to heightening climate change advocacy social norms and providing information to increase their self-efficacy for climate change action. Finding the appropriate actions and training individuals in necessary skills to be effective and comfortable engaging in climate actions is critical to address the well-being of our planet’s ecosystem and inhabitants in a timely fashion.

## Figures and Tables

**Table 1:** Demographic Characteristics and Responses to Climate Change Items from 586 Participants who completed the MTurk Surveys at Waves 1 and 4.

Demographic Characteristics	N 586
Age (years), mean (SD)	39.6 (11.7)
Female	336 (57.3%)
Race:	
White	475 (81.1%)
Black	37 (6.3%)
Other	74 (12.6%)
Undergraduate degree or higher	326 (55.6%)
Annual income > $60,000	267 (45.6%)
Political ideology ^1^	3.0 (2.0–5.0)
**Climate Change Actions**	
Participant would post materials online about climate change	276 (47.1%)
Participant would contact their state legislators about climate change	269 (45.9%)
Participant would talk to their friends about climate change	339 (57.8%)
**Climate Change Attitudes and Social Norm**	
How concerned are you about climate change	
Great deal	235 (40.2%)
Quite a bit	148 (25.3%)
Some	120 (20.5%)
Very little or none	81 (13.9%)
The future of many young people will be much worse due to climate change	
Strongly agree	181 (30.9%)
Agree	216 (36.9%)
Neither agree nor disagree	108 (18.4%)
Disagree	43 (7.3%)
Strongly disagree	38 (6.5%)
I can do nothing about climate change	
Strongly agree	41 (7.0%)
Agree	82 (14.0%)
Neither agree nor disagree	86 (14.7%)
Disagree	249 (42.5%)
Strongly disagree	128 (21.8%)
Most of my friends would disapprove if I were to post things on social media on climate change	
Strongly agree	10 (1.7%)
Agree	35 (6.0%)
Neither agree nor disagree	121 (20.6%)
Disagree	227 (38.7%)
Strongly disagree	193 (32.9%)
Most of my friends think that climate change is a major issue	
Strongly agree	111 (18.9%)
Agree	223 (38.1%)
Neither agree nor disagree	139 (23.7%)
Disagree	65 (11.1%)
Strongly disagree	48 (8.2%)
If I were to talk to most of my friends about the impact of climate change, they would be uncomfortable	
Strongly agree	7 (1.2%)
Agree	54 (9.2%)
Neither agree nor disagree	121 (20.6%)
Disagree	160 (27.3%)
Strongly disagree	214 (36.5%)
I tend to avoid news on climate change.	
Strongly agree	26 (4.4%)
Agree	47 (8.0%)
Neither agree nor disagree	73 (12.5%)
Disagree	226 (38.6%)
Strongly disagree	214 (36.5%)
With everything else going on in the world, I don’t have much interest in climate change	
Strongly agree	55 (9.4%)
Agree	82 (14.0%)
Neither agree nor disagree	173 (12.5%)
Disagree	218 (37.2%)
Strongly disagree	158(27.0%)

^1^ Scale goes from 1-very liberal, 2-liberal, 3-slightly liberal, 4-moderate, 5-slightly conservative, 6-conservative, 7-very conservative.

**Table 2:** Predictors of 3 Forms of Climate Change Communication Behaviors from 586 Participants who completed the MTurk Surveys at Waves 1, and 4.

	Participant Would Post Climate Change Materials ^1^		Participant Would Contact State Legislators about Climate Change ^1^		Participant Would Talk to Friends about Climate Change ^1^	
	OR (95% CI)	aOR (95% CI)	OR (95% CI)	aOR (95% CI)	OR (95% CI)	aOR (95% CI)
Climate change social norms measurement ^2,3^	**1.53 (1.40, 1.67)**	**1.27 (1.14, 1.41)**	**1.44 (1.33, 1.57)**	**1.12 (1.01, 1.24)**	**1.62 (1.48, 1.78)**	**1.27 (1.13, 1.42)**
Climate change avoidance measurement ^4,5^	**1.66 (1.49, 1.85)**	**1.27 (1.10, 1.46)**	**1.81 (1.61, 2.03)**	**1.40 (1.21, 1.63)**	**1.95 (1.73, 2.19)**	**1.41 (1.22, 1.64)**
Perceived self-efficacy to address climate change ^6^	**1.72 (1.47, 2.01)**	**0.99 (0.80, 1.23)**	**1.97 (1.67, 2.33)**	**1.14 (0.92, 1.41)**	**2.34 (1.96, 2.78)**	**1.42 (1.13, 1.78)**
Climate change will have negative impact on future ^6^	**2.61 (2.14, 3.20)**	**1.54 (1.19, 1.99)**	**2.70 (2.20, 3.32)**	**1.63 (1.26, 2.13)**	**2.99 (2.43, 3.68)**	**1.67 (1.28, 2.20)**
Race						
Non-Hispanic white	Ref	Ref	Ref	Ref	Ref	Ref
Non-Hispanic Black	1.09 (0.56, 2.13)	1.10 (0.51, 2.37)	1.15 (0.59, 2.24)	1.52 (0.70, 3.26)	1.61 (0.79, 3.28)	2.13 (0.91, 4.99)
Other ^7^	1.15 (0.70, 1.88)	0.82 (0.45, 1.49)	1.15 (0.70, 1.87)	0.92 (0.52, 1.65)	1.27 (0.77, 2.10)	0.95 (0.51, 1.78)
Political ideology ^8^	**0.63 (0.56, 0.70)**	0.81 (0.71, 0.92)	**0.66 (0.60, 0.73)**	**0.85 (0.74, 0.96)**	**0.66 (0.60, 0.73)**	0.90 (0.78, 1.04)
Age (years)	0.99 (0.97, 1.00)	1.00 (0.98, 1.02)	1.00 (0.98, 1.01)	1.01 (0.99, 1.03)	0.99 (0.98, 1.00)	1.01 (0.99, 1.03)
Sex						
Male	Ref	Ref	Ref	Ref	Ref	Ref
Female	1.39 (1.00, 1.93)	1.34 (0.89, 2.02)	1.21 (0.87, 1.68)	1.09 (0.73, 1.64)	1.14 (0.82, 1.59)	0.93 (0.60, 1.45)
Education level						
Some college or less	Ref	Ref	Ref	Ref	Ref	Ref
Undergraduate degree or higher	0.96 (0.69, 1.33)	0.85 (0.56, 1.29)	1.07 (0.77, 1.48)	0.75 (0.49, 1.13)	0.85 (0.61, 1.19)	**0.53 (0.33, 0.83)**
Annual income						
Less than $60,000	Ref	Ref	Ref	Ref	Ref	Ref
$60,000 or more	**0.56 (0.40, 0.78)**	**0.52 (0.35, 0.79)**	1.10 (0.79, 1.52)	1.37 (0.91, 2.06)	0.81 (0.58, 1.13)	0.90 (0.58, 1.41)

^1^ Logistic regression with “no” as reference group; ^2^ Climate change social norms scale: 3 (low)-15 (high); ^3^ Calculated by summing the responses to the following items: (1) If I were to talk to most of my friends about the impact of climate change, they would be uncomfortable, (2) Most of my friends would disapprove if I were to post things on social media on climate change, (3) Most of my friends think that climate change is a major issue. Response options are: 1-strongly agree, 2-agree, 3-neither agree nor disagree, 4-disagree, 5-strongly disagree; ^4^ Climate change avoidance scale: 2 (tends to avoid)—10 (does not avoid); ^5^ Calculated by summing responses to the following items: (1) I tend to avoid news on climate change, and (2) With everything else going on in the world, I don’t have much interest in climate change. Both items have the response options: 1-strongly agree, 2-agree, 3-neither agree nor disagree, 4-disagree, 5-strongly disagree; ^6^ Responses options: 1-strongly agree, 2-agree, 3-neither agree nor disagree, 4-disagree, 5-strongly disagree; ^7^ Includes Hispanic, Asian, Mixed, Other; ^8^ 1-very liberal, 2-liberal, 3-slightly liberal, 4-moderate, 5-slightly conservative, 6-conservative, 7-very conservative. Bold indicates *p* < 0.05.

**Table 3:** Chi-square statistics values for the associations between climate change communication actions and social norms (N = 586) ^1^.

Climate Change Communication Actions			
Social Norm Variables	I would be willing to post materials online about climate change	I would be willing to contact my state legislators about climate change	I would be willing to talk to my friends about climate change
Most of my friends think that climate change is a major issue.	107.5	104.2	135.0
If I were to talk to most of my friends about the impact of climate change, they would be uncomfortable.	32.7	27.7	42.8
Most of my friends would disapprove if I were to post things on social media on climate change.	94.9	57.9	89.0

^1^ Response options: 1-strongly agree, 2-agree, 3-neither agree nor disagree, 4-disagree, 5-strongly disagree. All Chi-square values highly significant, *p* < 0.001.

## Data Availability

Data is available from the first author.
